# *“You’ve got to look after yourself, to be able to look after them”* a qualitative study of the unmet needs of caregivers of community based primary health care patients

**DOI:** 10.1186/s12877-018-0962-5

**Published:** 2018-11-12

**Authors:** Kerry Kuluski, Allie Peckham, Ashlinder Gill, Jasleen Arneja, Frances Morton-Chang, John Parsons, Cecilia Wong-Cornall, Ann McKillop, Ross E. G. Upshur, Nicolette Sheridan

**Affiliations:** 1 Bridgepoint Collaboratory, Lunenfeld-Tanenbaum Research Institute, Sinai Health System, Toronto, Canada; 2 Institute of Health Policy, Management and Evaluation, Dalla Lana School of Public Health, University of Toronto, Toronto, Canada; 3 Epidemiology Division, Dalla Lana School of Public Health, University of Toronto, Toronto, Canada; 4 School of Nursing, Faculty of Medical and Health Sciences, University of Auckland, Auckland, New Zealand; 5 Division of Clinical Public Health, Dalla Lana School of Public Health, Toronto, Canada; 6 Department of Family and Community Medicine, University of Toronto, Toronto, Canada; 7 Centre for Nursing and Health Research, School of Nursing, College of Health Te Kura Hauora Tangata, Massey University, Albany, New Zealand

**Keywords:** Caregivers, Primary care, Qualitative, Canada, New Zealand

## Abstract

**Background:**

There is growing reliance on unpaid caregivers to provide support to people with care needs. Integrated care approaches that aim to coordinate primary care with community care known as community based primary health care (CBPHC) has been a key policy initiative across health systems; however most attention has been paid to the needs of patients and not caregivers. The objective of this paper was to explore the unmet needs of caregivers of older adults with complex care needs receiving CBPHC.

**Methods:**

This qualitative descriptive study entailed one-to-one interviews with 80 caregivers from Canada and New Zealand where roles, experiences and needs were explored. Interview text related to unmet need was reviewed inductively and core themes identified.

**Results:**

Three themes were identified across CBPHC sites: unrecognized role; lack of personal resources; and no breaks even when services are in place.

**Conclusions:**

To support caregivers, models of care such as CBPHC need to look beyond the patient to meaningfully engage caregivers, address their needs and recognize the insight they hold. This knowledge needs to be valued as a key source of evidence to inform developments in health and social care.

**Electronic supplementary material:**

The online version of this article (10.1186/s12877-018-0962-5) contains supplementary material, which is available to authorized users.

“…first of all you’ve got to look after yourself, to be able to look after them, and that’s where I fell down…I went crashing down myself, realising that I couldn’t look after myself. I mean, I couldn’t look after my mum, so I called out for help. Fortunately that help arrived, but boy it was the last of the light brigade arrived. They just got there in time.”

(New Zealand, Case 2, Caregiver-22)

## Background

There is growing reliance on ‘informal’ caregivers to support people with care needs. Caregivers’ work has been described as “largely invisible, unfairly distributed, with a huge effect on their opportunities in life” [1] and typically unpaid. An increasing demand for ‘informal’ care is part of a system-wide problem as health care systems strive to improve care that is better aligned to people’s needs and contain growing costs [2]. These goals create further impetus to draw on, as well as support the role of ‘informal’ caregivers (referred to herein as caregivers).

Caregivers engage in a range of unpaid activities with people (typically family) who experience anything from minor, episodic impairments to ongoing complex conditions that require full time support [3]. Activities include care coordination, assistance with activities of daily living (toileting, dressing, eating), daily living tasks (such as grocery shopping, paying bills, transportation, housekeeping), and complex treatments (such as wound care, and IV therapies). Even when patients access publicly funded services, many caregivers provide high volumes of care concurrently [4]. While caregiving has been shown to be associated with personal satisfaction and fulfillment [5], there are a number of well documented personal, social, and economic impacts, such as, time away from paid employment and leisure activities, interruption of personal relationships, and effects on physical and mental health [6–10]. Caregivers have many unmet needs related to: managing patients health [11], knowing what to expect [12] making decisions [13]; and accessing respite care [14], personal care and lighter supports (companion care and preparing meals) [11]. Caregivers have also indicated an absence of someone they can count on to organize care and are seeking better follow-up when complaints arise [15]. The disruptive nature of services or perception of poor quality of care may result in caregivers refusing services, which further perpetuates unmet patient and caregiver needs [16].

While the challenges for caregivers are well established, health systems continue to prioritize the medical aspects of patients [17]. Given high rates of caregiver burnout, poor health outcomes for patients and caregivers and broader economic implications of providing care (e.g., lost time from work and other opportunity costs for caregivers), policy goals for improving care quality must afford greater attention to caregivers; particularly their unmet needs to ensure interventions to address their needs are incorporated alongside patients. We observe two key gaps in the literature. While there is a growing body of evidence of the experiences of caregivers of older adults who have complex care needs (e.g., multimorbidities combined with social needs due to low income, isolation, language barriers, etc.) there are few studies on caregiver unmet needs in a primary care setting particularly among ethnically diverse caregivers [16]. Second, it is unclear if integrated models of care (i.e., services that are coordinated or linked together), designed for patients, have spillover effects for caregivers by way of reduced stress and improved quality of life. While our study engages with ethnically diverse caregivers, our study is designed to only provide some insight into the second knowledge gap (spillover effects of integrated care models for caregivers).

The objective of this paper was to identify the unmet needs of caregivers. These caregivers were caring for patients with complex care needs receiving community based primary health care (CBPHC); a type of integrated care model that combines primary care services with care in the community. While we did not specifically endeavor to explore unmet needs that stem from the CBPHC models alone, we examined unmet needs, in general, which can have implications for the delivery of CBPHC in the future.

We examined CBPHC models in two jurisdictions, Ontario (Canada) and New Zealand (NZ) that were selected for their reputations of innovation and for serving high needs populations. Many of the models were serving culturally diverse (CAN, NZ), immigrant (CAN) or indigenous populations (NZ). The models were selected following a literature review and expert consultation [18]. While a more comprehensive comparison of Canada’s and NZ’s health systems are provided elsewhere, [19] we highlight here that both countries have been reforming primary physician services from solo to group based models with a greater role for nurses, nurse practitioners and allied health professionals. Some primary care models extend further into the community sector (home and community supports) and these models—which we refer to as CBPHC, are varied both across and within each country. In both countries, the CBPHC sites featured in this study did not necessarily include all services needed by patients and their families therefore their unmet needs reflect their experiences with a range of other services including specialist services, homecare, hospital care, and community supports (e.g., transportation, food banks). This in and of itself speaks to the limitations of systems that are not wholly integrated.

## Methods

The methodological orientation of the study was qualitative description, an appropriate method for research examining the “who, what, and where” of phenomena that are poorly understood [20] Consistent with this methodology no theory was used a priori to guide the analysis nor was a theory developed from the results (as is the case with grounded theory) [21]. We used inductive thematic analysis as the method, appropriate for an exploratory study. The study entailed semi-structured interviews with 80 caregivers in six CBPHC sites located within Ontario, Canada and NZ (Additional file 1).

A convenience sampling technique was used. Adult caregivers were eligible to participate if they were providing unpaid support for an older adult (50 years+) who was linked to one of the CBPHC sites and required ongoing attention and multiple services. Ongoing engagement with admin staff ensured that they were reaching out to caregivers as per our eligibility criteria. The 50+ cut-off was recommended by the site leads who serve more vulnerable populations as the aging process starts much sooner. While all caregivers were currently providing care at the time of the study, some also had previous caregiving experiences and/or were caring for multiple people simultaneously. Administrative and front line staff at each site approached eligible caregivers (in person or by phone), and sought approval to have a researcher contact them by telephone to explain the study and book an interview. A researcher followed up with interested caregivers to explain the study and seek consent. Interviews often took place in the caregivers’ home or at a location of their choice. Ethics approval was obtained from the University of Toronto (Protocol 128,263) and University of Auckland (Protocol 013071). Interviews were conducted, one-to-one, in person, between May 2015 and October 2016 and took between 30 and 60 min to complete. Two researchers conducted the Canadian interviews and four researchers conducted the NZ interviews (though, one researcher per country conducted most interviews). All data collectors were trained in qualitative methods and had previous experience conducting interviews with older adults and caregivers. Two of these data collectors were PhD Candidates and the others were Professors and clinicians. The researchers had no relationship to the participants. At the beginning of each interview the interviewer briefly introduced his/her self and explained the objectives of the study [“to better understand their experiences”]. No bias or personal interests were reported by the interviewers. No repeat interviews were conducted.

Caregivers were asked questions about their roles and health system experiences. Background characteristics (Table 1) were collected through close ended questions. In addition to fixed response categories the interview included several open-ended questions (which informed this paper). The interview guide (Additional file 2) was pilot tested in both study jurisdictions. Although the interview guide contained many structured questions, it was used as a guide only. The order of questions shifted in accordance with the care recipient responses and the style of the interview allowed participants to elaborate, move in new directions and provide additional context. Within the various case sites, saturation of broad thematic findings was seen between the first 6–13 interviews, approximate to the number of interviews conducted per site. The interviews were audio-recorded and transcribed verbatim. Transcripts were not returned to participants for comment. Twelve caregivers were non-English speaking and these interviews were conducted with a translator and the English translation was transcribed.

**Table 1:** Caregiver demographic information

Variable	Total Sample N	Frequency n	Percentage
*Translated Interviews*	80	12	15
Cantonese, Mandarin		11	
Italian		1	
*Location*	80		
Canada		39	49
New Zealand		41	51
*Age*			
25–34		1	1.3
35–49		14	18
50–64		34	43
65–74		17	21
> 75		14	18
*Sex*	80		
Female		66	83
Male		14	18
*Ethnicity*			
East Asian (Chinese, Vietnamese)		26	33
European (New Zealand, Italian, Greek)		22	28
Maori		19	24
Canadian (Caucasian)		5	6.3
Caribbean (Jamaican, Guyanese)		4	5
South Asian (Indian, Pakistani)		3	3.8
European and Maori		1	0.13
Health Conditions of Care Recipient			
Average Number of Health Conditions		5	
*Living Arrangement*	80		
Living with Client		59	74
Not living Client		21	26
*Relationship to Care Recipient*	80		
Child		41	51
Spouse		32	40
Other		7	8.7
*Sex of Care Recipient*	80		
Female		48	60
Male		32	40
*Chronic Conditions Managed by Caregivers*	80		
Coronary Obstructive Pulmonary Disease		40	50
Cancer in the last 5 years		39	49
Asthma		38	48
High blood pressure		36	45
Ischemic Heart Disease		36	45
Diabetes		35	44
High Cholesterol		32	40
Stroke		30	38
Arthritis		28	35
Other (Dementia, Alzheimer’s, Cataracts, Hearing Impairment)		26	33
Chronic Pain		24	30
Mental Health (Anxiety, Depression)		24	30
*Support Provided by Caregiver*	80		
Personal care		45	56
Day-to-day assistance		67	84
Household chores		69	86
Additional support: Companionship, Decision making		71	89
*Caregivers participating in paid employment*	80	21	27
*Financial Burden* ^a^	77		
Finances adequate to pay for health needs		36	47
Caregiving put financial strain on the family		26	34
Difficult to pay for patient’s needs		41	53

^a^Caregiver reported a response of *Agreed* or *Strongly Agreed*

Six team members from both jurisdictions reviewed a sub-set of interviews [the initial interviews completed] and a codebook was created over several meetings where content and definitions were deliberated. While the researchers did not specifically ask about unmet needs in the interview (aside from a couple of open ended questions that probed in that direction); this particular category of data became apparent after reviewing the first set of interview transcripts and thus all interviews were coded for ‘unmet need’. Two researchers used the codebook to code the full set of interviews checking each other’s work after the first few interviews to ensure consistency in approach (queries were organized using memos) and referred to during subsequent discussions to iron out discrepancies. Qualitative data analysis software (NVivo 10) was used.

The code “unmet need” was extracted from the codebook, a node report generated and analyzed inductively by two team members. The analysis involved multiple readings and interpretations of the data in order to create core logic categories that were derived by dividing text into similar meaning units [22]. For example, initially text was divided into broad categories: missing services; poor quality of care; difficulty accessing care; not enough services; willingness to use care. An in-depth round of coding followed with additional team members to organize these categories into themes and reduce conceptual overlap. This stage entailed reading through all text in each category and the full node report again to make sure important text was not missed. Multiple coders, consensus meetings, and prolonged engagement enhanced trustworthiness of the data. The broader team was consulted to verify that the themes were interpreted correctly, and where needed, additional context was added (theme descriptions). The findings were not returned to participants for comment.

A total of 80 caregivers completed interviews after 114 were approached to participate. The most common reason for non-participation was not being able to reach caregivers or caregivers refusing to participate due to busy schedules.

The majority of caregivers were over the age of 50, female, and ethnically diverse. Most caregivers were caring for a parent or spouse/partner with whom they resided. Overall, the samples within both Canada and NZ were ethnically diverse with proportionately more Chinese caregivers in Canada; and all Maori participants from NZ. All of the translated interviews were from the Canadian sample. Care recipients had an average of 5 health conditions. Caregivers provided support with personal care, instrumental activities of daily living (IADLs), companionship, and decision-making. Many experienced financial burden. [see Table 1].

## Results

The qualitative findings of unmet need were organized into three themes: unrecognized role; lack of personal resources; and no breaks even when services are in place. These themes were present in both jurisdictions across the CBPHC sites.

### Unrecognized role

Caregivers generally felt unrecognized in their role. They wanted to be acknowledged for the care they provided to the patient, the insight they had of patients’ needs as well as have their own need acknowledged. Recognizing caregiving as a form of valued citizenship was emphasized in one case. This caregiver contended that government had a central role to play in supporting caregivers, suggesting that an allowance was not the only assistance that might be provided.

*“… I don’t believe for one minute that every caregiver needs to be paid, but every caregiver needs to have a response from the government that allows for some flexibility for their lives while they are doing a first class job in caring for a loved one that, put in hospital, would cost the government a huge amount of money.”* (NZ, Case 3, Caregiver-9)

Caregivers in this study were often the only consistent form of support for the care recipient as they moved between settings and providers. Given the long-standing relationship between caregivers and care recipients there was often a great deal of specialized knowledge of patient need, preference and capacity that exceeded formal provider knowledge. This recognition of patient preferences and needs, regarding medical tasks, ranged from resuscitation orders to the recognition of triggers in patient behavior that signaled that something was wrong. Caregivers felt that the care team did not always recognize these integral pieces of knowledge and, in some cases they had to be diligent about clearing up misunderstandings.

Caregivers themselves have care needs and this was not always picked up by the care team. A caregiver in one case was not recognized as someone with a mental health issue. When he became very ill, a friend was able to connect him to needed mental health care. Initially, providers who worked with his mother were unable to recognize his health challenge:

*“…everyone around here just saw the two of us getting on. I don't think they quite realised just how bad I had got until I wound up being taken away…You could say that people like me in my situation tend to fall through the cracks.”*(NZ, Case 2, Caregiver-22)

While most caregivers did not question the value of their caregiving or express a desire to withdraw, they sensed that their presence mitigated state funded support:

*“…at the end of the day, not every whanau [i.e., family] has got that assistance. And particularly for people living way up in the coast, any home help or any caring is very difficult for them […] there seems to be an attitude coming from those from the bureaus that,” “If you are doing it, well, why do we need to intervene?”* (NZ, Case 3, Caregiver-9)

Some caregivers expressed that caregiving was “just what you do,” or expressed a lack of confidence in the health care system which limited their engagement. Others felt that their needs fell outside the realm of what health care providers *could* do:

*“I am very happy with how I’m treated and asked how am I, and is there anything they can do? It’s like what could you do? You know, it’s a financial burden, it’s a social burden, it’s an emotional burden. And there's nothing somebody as a professional coming in can help with that really.”* (Canada, Case 2, Caregiver-8)

### Lack of personal resources

Caregivers made significant sacrifices as they provided care and received little, if any, financial, informational and educational resources to support them. Oftentimes, caregivers drew on their own finances to both provide and access care. Financial strain was amplified by taking time from (or permanently leaving) paid employment to provide care. Although caregivers in Canada and NZ may be eligible for protected (typically unpaid) time from work, and tax breaks, the eligibility and time restrictions often rendered the supports insufficient (or irrelevant if the caregiver is not in the paid workforce):

*“There has to be some sort of financial help for people who are caring for their families at home. And I’m going to be profane here; the caregiver allowance that you can claim on your income tax is bullshit. I don’t have any income.”* (Canada, Case 2, Caregiver 8)

Caregivers expressed frustration at the assumptions made by providers about what they were capable of, or willing to do:

*“…he [physician] keeps on saying that it’s like our responsibility. Like [homecare] only can help so much […] We cannot even leave her. It’s not easy. So he said, “Oh, I guess you have to hire somebody to watch your mom.” I said, it's easy to say but where are we going to get the money?”* (Canada, Case 1, Caregiver-27)

When available, financial support was difficult to access, and required onerous paperwork. Given the time involved in applying for support some caregivers “simply gave up.”

Ongoing workforce interruptions were noted. In the case detailed below, the caregiver used her vacation days to care for her Mom:

*“It’s all my vacation days or I take it as unpaid. Which to me I think is ridiculous […] I know that the government has something in place that they’ll pay you for sick leave with regards to, you know, God forbid you’re dying of cancer. But what about people that… You know, my mom’s constantly into the hospital.”* (Canada, Case 3, Caregiver-11)

The following caregiver expressed gratitude for having protected time from work after her mother died:

*“I suffer with anxiety after mom passed, and depression myself, and I took six weeks off. Where would I have taken six weeks off in another job? So I feel that this government should make some allowances […] there should be more in place.”* (Canada, Case 2, Caregiver-13)

If caregivers had the means to pay, they “topped up” publicly funded services by purchasing additional care, usually homecare, out-of-pocket because the portion of care that was publicly funded was not available in the desired quantity and scope. The caregiver featured here had paid supports in place for her mother allowing them to live separately, until they no longer had resources available to do so:

*“So the situation changed about 3 years ago…And I had caregivers come, private. I paid for. And then of course the money ran out and I had to make the decision to move in and take care of her myself.”* (Canada Case 3, Caregiver-12)

After depleting her financial resources, this caregiver came to rely on her mother’s pension for living expenses. She was concerned that if her mother transitioned to a long-term care facility the monies would get redirected to pay facility costs and leave her in a precarious financial and housing situation.

Caregivers incurred expenses from purchasing equipment including hospital beds, ramps and grab bars. Health supplies to manage incontinence (pads, catheters), wound care, and medicines generated significant costs. In a few cases, these medical supplies were partially subsidized by the government, the care organization providing them, or by district nurses and nurse practitioners (in NZ). A Canadian caregiver suggested that it would be useful to have an “aid to daily living” fund to support the cost of supplies or a ‘co-op’ to access and share resources with other caregivers. Even when subsidized, some level of co-payment or rental fee was usually required:

*“We’ve been paying a lot of our stuff for that out of our own pockets, by getting pull ups for her, pads.”* (NZ, Case 2, Caregiver-19)

Some caregivers experienced severe hardship and were themselves older adults with chronic conditions and faced a double burden of costs which impacted access:

*“He's been without medication, and so have I, because we haven't been able to afford it.”* (NZ, Case 2, Caregiver-21)

Educational support and training to help with present and future needs was identified as lacking. Such preparation and education was seen as a matter of necessity to safely care for the patient and stay in control of a situation that was often unpredictable. Caregivers expressed that it was important to know how and where to seek supports particularly in the earlier stages of caregiving, to avoid unnecessary, longer term hardship.

Caregivers wanted additional support when managing complex symptoms of care recipients. In this first example, the caregiver was receiving many supports from providers who were all struggling to manage a severe wound:

*“…she hasn’t been up for 6 weeks at the moment. She wanted to get up yesterday and I said the wound opens, it closes […] as soon as she gets up and puts pressure on it opens up again. So I don’t know what, what to do.”* (NZ, Case 2, Caregiver-18)

Another caregiver described what it was like managing incontinence:

*“Nobody tells you that you’re going to need 50 nighties because they’re peed every day, 5 times a day. And if you don’t do the washing every day, what are you going to have? Are you going to have 4 underpads that are dirty? And you need 6 sets of sheets, and they’re always going to stink.”* (Canada, Case 2, Caregiver-8).

Finally, several caregivers were caring for family members with dementia which created a unique set of challenges due to changing moods and behaviors:

*“Because I’m not trained, it’s trial and error. And then because she’s getting dementia worse and worse now. She’s hiding things, putting things away. You just cannot find it or help her to find it. And she gets frustrated and I get frustrated.”* (Canada, Case 1, Caregiver-26).

### No breaks even when services are in place

All patients were receiving an array of services—in addition to care from their CBPHC site resulting in various combinations of primary and specialist care, homecare, community supports and hospital care. As patients used these services, caregivers continued to play an active role by coordinating care. Dealing with deficits in the formal care system was frustrating for caregivers because they had to *“be on top of everything”* and *“jump through hoops”* to get what they needed. Even if patients had a supportive primary care provider they still had to coordinate other services:

*“Yeah, it is, because everything, you have to go through people. You know, you’ve got to get referrals from specialists, referrals from doctors …”* (NZ, Case 3, Caregiver-8).

Long waits, unpredictable arrival times of homecare providers, and poor communication with front-line staff meant that caregivers often had to step in to do the job:

*“I asked them to come at noon because she had a 1:00 appointment. So I changed her bed because I had to dress her and everything… The girl comes in at 1:00.” I said, “Did the office not tell you?” She said, “No, the office didn’t tell me anything.”* (Canada, Case 3, Caregiver-8)

Caregivers had to repeatedly provide information about patients’ needs, preferences, and treatment regimens, particularly to different homecare providers due to high turnover of staff:

*“So like one person would come and they would have a schedule, and you would know they would come at this time. Then they would change it, and you wouldn't know. Then the other person comes, and they don't have a clue what they're doing. So it’s me now having to educate this new person.”* (Canada, Case 3, Caregiver-8)

Care recipients typically felt most comfortable when they received support from a familiar provider. This is particularly important for people with cognitive decline where early intervention and continuity of care is critical to the acceptance of care. However, continuity of care is a major challenge in the homecare sector in both study jurisdictions. The patient may resist care when someone new or unfamiliar steps in, particularly when offering personal care activities. At times patients resisted care so the onus of responsibility fell to the caregiver:

*“I was lucky, I have one [personal support worker [PSW]] that came the first day when she was sick. She got used to her. She’s fine. I have so much trouble when she goes away on holiday, when somebody else comes in. [My mother is] picky, she will not, she will not poo. She doesn’t do anything because [the PSW] a stranger to her.”* (Canada, Case 2, Caregiver-11)

Caregivers pointed to the limitations of services which primarily focused on personal care and activities of daily living, and in limited quantities. Bathing, toileting and wound care were among the types of care that caregivers had to step in to help provide, given the unpredictability of the timing and frequency with which such needs occur.

A lack of certain services added more to the caring load. While each situation varied, many times, accessing help with important but non-medical care like social visits, housekeeping, and assistance with meals, night care, home maintenance and other IADLs fell outside the scope of publicly funded service entitlements.

When a caregiver was asked what services might help her she pointedly stated:

*“What service? The only service I need is somebody to sit with my mother when I’m going out or do some running around.”* (Canada, Case 2, Caregiver-11)

Caregivers noted that the hours of care provided by the ‘formal’ system were inadequate to get a true break:

*“I would think the [homecare agency], if they provide to extend to weekends as well, 7 days. It’s more consistent. Then her linens, like bedsheets and everything [would get washed]… It’s just caring for her is quite time-consuming […]..it would free me up. At least she’s safe. Not only just taking care of her bathing, you know, sometimes just chatting with her. That also opens her up. You know, it’s a good medicine.”* (Canada, Case 1, Caregiver-26)

Programs for caregivers, such as respite care, are meant to provide a break, allowing the caregiver time to rejuvenate, get things done, and step away from caring duties for a period of time. Unfortunately these types of programs did not always meet the desired expectation:

*“But I didn't really want her in there anyway. Because it was no rest for me! If she’s in respite care… when she was in hospital, I would go up there 2 or 3 times a day. And if she’s in respite care, I would be there as long as she was! Pointless!”* (NZ, Case 1, Caregiver-29)

Even during hospital stays, caregivers assumed a continuous role. In this case, the caregiver felt that formal care providers were less attentive because she was there.

*“It’s about kind of managing them as well, because quite often I find there they will leave us all day by ourselves. Because I'm there, and they just think I’ll do everything so they don’t have to.”* (NZ, Case 3, Caregiver-12)

## Discussion

The study engaged 80 caregivers of various ages and ethnicities, caring for older adults with complex care needs. Three common themes were synthesized and explored which were relevant to caregivers across multi-language, linguistic and ethnic groups, making an important contribution to the literature and serving as an anchor for future policy direction.

Our findings are consistent with previous evidence on caregiver unmet need, reinforcing that caregivers play an integral role in society, providing significant volumes of support, often, at the expense of their own goals, priorities and health. They become the eyes and ears of patients, navigate complicated health and social care systems, guide care providers on how best to deliver care in line with the preferences of their loved ones, and fill important gaps. Caregivers in this study paid significant costs in time, effort and finances despite their care recipients being attached to a wide range of health care supports and from models of care that had reputations for being innovative [18].

Previous research has shown that caregivers assume considerable responsibility for the care recipient, and report guilt, and strained personal and social lives due to the physical and emotional work of caregiving [23, 24]. Similarly, caregivers of patients with multiple chronic conditions in primary care reported feeling overwhelmed, drained and “split into pieces” [16].

### Unrecognized role

Support for caregivers is necessarily rooted in an overall awareness of the value of the caring role. Some caregivers became resigned to their role, lost faith in, or had defined expectations of what the health system could deliver. Recall the caregiver who was happy with how she was treated by health care staff only to go on and articulate the emotional, social and financial burden of care. She noted that these challenges—which extended beyond the medical aspects of care-- were not things that professionals could help with. Importantly it seems that the expectations caregivers have about what the health care system can offer them, may shape their assessment of it and levels of engagement.

Related to caregiver recognition, the NZ *Health of Older People Strategy* [25] aims to improve caregiver resilience, improve supports and reduce work related barriers to care. The Whānau Ora (i.e., family health in Māori) has the goal of empowering local communities in all aspects of life. Likewise, the NZ Caregivers Strategy [26] is designed to protect caregiver health, helping them achieve balance with employment, and recognize their contributions. Two CBPHC sites in NZ served primarily Maori indigenous populations and were strongly rooted in Whanau/family values. Unmet needs appeared to persist, perhaps because caregivers were interacting with many other parts of the health and social care systems which do not necessarily embed the same philosophy of care, at least not to the same degree as the CBPHC site. This calls for the importance of the role of care coordination [27], as a mechanism to support caregivers who are interacting with disparate parts of the health system, within and alongside models of care like CBPHC.

At the provincial level in Canada, there are some examples of caregiver recognition, including, for example, Manitoba’s Caregiver Recognition Act where the Minister responsible regularly consults with caregivers and reports progress at defined intervals. Ontario is also looking to formally recognize caregivers through similar legislation (Bill 138).

The Change Foundation, a policy think tank in Ontario Canada conducted a province-wide consultation with caregivers and, similar to our findings, found that caregivers did not always recognize themselves as caregivers nor did formal providers [28]. Many caregivers in our study expressed that caring was “just something that you do” as a partner, family member, friend or neighbor.

To recognize and support caregivers, the UK has developed a caregivers assessment of capacity and needs [29] to which all caregivers are entitled. The assessment is an opportunity to collect population level caregiver trends, identify and respond to service needs and identify gaps. Despite this development, caregivers, in the UK remain unaware of this entitlement.

### Lack of personal resources

Caregivers emphasized the ongoing financial burden of care due to costs associated with taking time from work, purchasing medicines and supplies, and “topping up” services with privately purchased supports. While both countries offer financial relief for caregivers, the burden of mobilizing these supports becomes onerous. For example, the NZ Employment Relations Act (2000) entitles caregivers to flexible work hours. The Supported Living Payment supports non-spousal caregivers who provide the equivalent of residential or hospital care and the Caregiver Support Subsidy is available to unpaid full time caregivers of disabled persons. In Canada, the Employment Insurance Act (1996) entitles employees to Compassionate Care Benefits, ranging from 8 to 37 weeks of protected, unpaid leave, depending on the province, but it is limited to caregivers looking after someone who is near death. A number of tax credits for low-income caregivers, those of dependent children and veterans are also available [28, 30]. Caregivers in our study, as in other research, were often unaware of their entitlements, or did not have the time and capacity to figure out how to obtain these supports leading to unnecessary out-of-pocket expenses. Previous work has also shown that people have tried these types of supports, had a negative experience and stopped using it [31], pointing to a larger challenge of the *value* of these supports. Our study highlights the long-term implications of financial burden as shown in the case of the caregiver who depleted her financial resources to support her mother. When assessing the opportunity costs of caregivers, a long-term snap shot is required to capture the true financial impact of caregiving so that appropriate compensation can be provided.

Caregivers in our study were seeking guidance on how to manage health related issues, including complex symptoms like wound management, incontinence and dementia related behaviors. These activities were difficult to manage even when supports were in place. The realities of complex care management suggest that an ‘all hands on deck’ approach may be required with no immediate relief to paid or unpaid caregivers. Our study results mirrors a previous study [32] which found that having to manage multiple medications, assist with mobility, deal with challenging symptoms and manage poor appetite, navigate memory loss and confusion contributed to significant caregiver burden.

### No breaks even when services are in place

Caregivers’ frustration surfaced as they interfaced with the various services required by the care recipient. The services available were not always enough—not provided at the right time or at the right level of quality. This speaks to the ubiquity of the caregiver role.

Quite commonly, caregivers had to step in, fill the gaps, and monitor patients’ health status and needs, particularly when receiving homecare services, given high turnover of staff and the need to constantly reorient providers to routines. Some key services that would benefit caregivers were missing including, IADLs (meals, housekeeping, and transportation) and social visits for patients. Likewise, in a previous study, caregivers of older adults articulated a lack of companion care, overnight care, and respite care, in addition to help with housekeeping and meal preparation [11].

We provide five recommendations for health systems to consider in addressing unmet caregiver needs.

First, adopting the UKs Caregiver Assessment, or similar modality, is a necessary catalyst for understanding the needs of the caregiver and responding with appropriate resources. While some insight into the caregiver (the types of care they provide and capacity to continue) is assessed as part of the interRAI homecare tool [33] used internationally, important context is missing including their personal needs, level of engagement and location (co-habiting, living separate including in another jurisdiction). In the short term, a broader care assessment like the one in the UK can point to system gaps that need to be filled in order to meet caregiver needs. While a necessary first step, without subsequent action in response to caregiver needs, outcomes will not change [34], thus implementation of supports needs to follow any assessment. The primary care setting, where the patient is receiving care, may be a natural place to identify the caregiver and assess their needs both individually and as a dyad.

Second, minimizing out-of-pocket costs for caregivers requires greater access to medical supplies and home adaptations at a reduced cost, expanding universal access to medication (e.g., particularly in Canada where out of hospital medicine costs often falls to the individual). Both countries have baseline compensatory supports (but limited to certain types of caregivers). Improving these programs by providing access to a wider group of caregivers with clear pathways to access (so patients, caregivers and providers know where to look for information) may be an important upfront investment.

Third, as part of any CBPHC model, it is important to have access to ‘core’ providers beyond the primary care provider including a consistent homecare provider that the patient and family can get to know over time; providing an opportunity to develop trust and a set of shared expectations and goals. Having a consistent home based provider would eliminate having to re-orient routines, patient preferences and needs. Patients are also more likely to be responsive to providers if they are comfortable, allowing the caregiver to have a break or complete other tasks.

Fourth, our study findings point to specific service needs among caregivers including social visits for the patient so the caregiver can leave the home and complete tasks; flexibility in service hours; and respite care that is designed in a way that allow caregivers to have a break. Respite care may benefit from a co-design approach so caregivers can articulate what needs to occur (in terms of service types, duration and location) for them to feel supported.

Fifth, we need to increase the status of the knowledge that caregivers hold so it is recognized as evidential as opposed to anecdotal. Caregivers carry critical knowledge of the patient, their needs and preferences that can inform better quality of care for patients as well as for themselves. Capturing and valuing this knowledge is necessarily linked to reforms in care provider education so caregiver recognition and inclusion in care planning become embedded in practice.

## Strengths and limitations

The strength of this paper is the large number of culturally and linguistically diverse caregivers from two jurisdictions that are represented in the data. By targeting CBPHC that had reputations for delivering exemplar patient care we had a unique opportunity to start to unpack whether or not this transferred to a better experience for the caregiver. Since the study was not designed to uncover unmet needs, specifically in relation to these models, future research is required to probe specifically into what these types of models do for caregivers and their effects on meeting caregiver needs. What became clear in our study is that both patients and caregiver use care well beyond CBPHC and thus experiences in other sectors combine with experiences of CBPHC. Disentangling these experiences is difficult to do, nor should be expected. This is the reality of health systems that are not fully integrated. Our study, in essence, captures unmet needs in health systems generally and cannot be contained to the CBPHC experience. Regardless, developed health systems are focusing on providing higher quality care for *patients* but the evidence from our study suggests that caregivers are getting left behind. Our themes provide concrete directions for the resources that are required to enable a better caregiver experience. Another limitation of our paper arises from the translated interviews. Since the English translation was transcribed, important nuances in language and meaning may have been lost. Another limitation is our focus on *unmet* need. We do not illustrate what care looks like when needs *are* met which is equally important to inform future programming. There were examples in our interviews when caregiver needs were met but this often happened in concert with unmet needs. In order to conduct a deep exploration into unmet need, this paper does not share those important examples. Future analysis by our team will delineate what a positive caregiving experience entails, including the specific tasks, roles and functions of teams that enable this.

## Conclusions

Caregivers often do not feel valued and recognized, lack financial and educational support, and seldom get breaks even when publicly funded services are in place. Our findings suggest that any model of care will need to consider such systemic issues as identified in our study, and cross health and social care boundaries, to address the unmet needs of caregivers. Perhaps there is a role for CBPHC to consider these wide ranging needs and build them into models of care in the future. Models of care such as CBPHC need to look beyond the patient to meaningfully engage caregivers, address their needs and recognize the insight they hold. This knowledge needs to be valued as a key source of evidence to inform developments in health and social care.

## Additional files

Additional file 1: Description of Community Based Primary Health Care Sites. This file contains a description of each of the case study sites across Ontario, Canada and New Zealand that are represented in this study. Each of the caregiver participants were looking after a patient from one of these sites. (DOCX 38 kb)

Additional file 2: Caregiver Interview Guide. This file contains the interview questions used to collect information from caregiver participants. (DOCX 51 kb)
