# Supplementary material for: “You’ve got to look after yourself, to be able to look after them” a qualitative study of the unmet needs of caregivers of community based primary health care patients
Source: BMC Geriatr. 2018 Nov 12;18:275. doi: 10.1186/s12877-018-0962-5 (PMC6233534; doi:10.1186/s12877-018-0962-5)
Supplement: Supplementary file 1 — Description of Community Based Primary Health Care Sites. This file contains a description of each of the case study sites across Ontario, Canada and New Zealand that are represented in this study. Each of the caregiver participants were looking after a patient from one of these sites. (DOCX 38 kb) [file 12877_2018_962_MOESM1_ESM.docx]

Additional File 1: Descriptions of Community Based Primary Health Care Sites (referred to as ‘cases’)

| Canada | Case 1 | Not-for-profit organization comprised community services (meals on wheels, day programs, homemaking, supportive housing, etc.), a family health team (primary care model comprised of a multidisciplinary team) and a foundation. Originally designed for (and continues to predominantly serve) the Chinese population. Additional targeted interdisciplinary assessment and care (collaboration between primary care and community services) is provided for persons with complex care needs. Additional programming is provided through partnerships with external providers (e.g., homecare agencies and hospital). |
| --- | --- | --- |
|  | Case 2 | Partnership between a Community Care Access Centre (Ontario’s coordinating agency for home and community care) and a Family Health Team. A CCAC Care Coordinator is embedded in the Family Health Team and targets and coordinates care for frail older adults with complex care needs. Several types of services are offered (including home services, house calls from physicians and telemedicine). Partnerships with local hospital, Emergency Services and pharmacies provide patients with access to a common set of providers. |
|  | Case 3 | Community Health Centre with two satellite and two larger “hub” sites offering a full range of primary care and community support services geared to a clientele of mostly new immigrant and disadvantaged populations. The sites are co-located with services for new immigrants, legal clinics, community gardens, and other community supports with free spaces for members of the community to host events. |
| New Zealand | Case 1 | Includes urban and rural general practices, practice nurses, pharmacists, contracted service providers (e.g., homecare), allied health professionals, the Canterbury District Health Board, Primary Health Organizations and general practitioner groups. The Network also includes a leadership team and several work streams dedicated to service transformation and improvement. Specific programs/funding are dedicated to emergency department and hospital prevention, including programs that “pull” patients from hospital to home for short term intensive services, home medication management programs and care coordination. |

|  | Case 2 | A long-term condition management program provided within six general practices (consisting of a nurse and kaiawhina (community health worker) who visit patients in the home with the aim of improving access and coordinating both health and social care. The practices are mostly in rural areas and a high proportion of the population are Māori, low-income and older. Though designed to be culturally sensitive to Māori population, the program serves people from many ethnicities. Designed to improve access to services, the nurse and kaiawhina visit clients in their homes, coordinate needed services and typically discharge the client after 6 months. This service is offered in addition to regular primary care services in which the program is embedded. The medical record is housed at the general practice. |
| --- | --- | --- |
|  | Case 3 | The Trust delivers primary health care and public health services in the community within a rural catchment area to a largely Māori population. Services include whanau ora (family/whanau health navigators), Kaumatua/Kuia (elder) services along with public health services (cervical screening and immunizations), mobile nursing services, programs in schools, etc. The service is supported by a sole Whanau (family) navigator who links clients government agencies and specialist services as needed. Primary health care is delivered in clinics (suburban and rural) by a small multidisciplinary team or in the patient’s homes. Public health and social services are delivered in a wider range of venues (schools, and marae (traditional meeting places). |
