# Supplementary material for: “You’ve got to look after yourself, to be able to look after them” a qualitative study of the unmet needs of caregivers of community based primary health care patients
Source: BMC Geriatr. 2018 Nov 12;18:275. doi: 10.1186/s12877-018-0962-5 (PMC6233534; doi:10.1186/s12877-018-0962-5)
Supplement: Supplementary file 2 — Caregiver Interview Guide. This file contains the interview questions used to collect information from caregiver participants. (DOCX 51 kb) [file 12877_2018_962_MOESM2_ESM.docx]

Additional File 2: Interview Guide

Family Carer Interview Schedule

We want to better understand your experiences as a family carer of (name).

First topic: Personal details and carer information

*Let’s start with your age*

1. What age group are you in?

🞏 <25yrs 🞏 25-34yrs 🞏 35-49yrs 🞏 50-64yrs 🞏 65-74yrs 🞏 >74yrs

1. 🞏 Male 🞏 Female
2. Which ethnic group(s) do you belong to?
3. What languages can you hold a conversation in?
4. What country were you born in? If not Canada:
   1. When did you move here? Why did you move?
5. What is your highest level of education?
6. Are you currently in paid work? Yes / No
   1. If Yes: What type of work do you do?
   2. How many hours do you work each week?
7. How are you related to X?
8. Do you live in the same house? YES / NO
9. If YES: How many others live in the house with you and X? How are you related?
10. If NO: How many days each week do you visit X?

What distance do you live from X? How long does it take to get to X’s home?

What form of transport do you use to get to X’s place?

9. How long have you been caring for X?

10. Does anyone else help with X’s care? YES / NO

1. If YES: Please explain
2. What are your main challenges in caring for X?
3. Has a doctor, or another person, ever told you that X has:

🞏 High blood pressure

🞏 High cholesterol

🞏 Diabetes

🞏 Ischaemic heart disease

🞏 Stroke

🞏 Cancer in the last 5 years other than skin cancer (not melanoma)

🞏 COPD

🞏 Asthma

🞏 Arthritis

🞏 Chronic pain

🞏 Diagnosed mental health disorder

🞏 Other, please state:

Second topic: Personal perspectives on care

*The next questions ask about your experiences, beliefs and perspectives in providing care*

|  | Strongly Agree | Agree | Neutral | Disagree | Strongly Disagree |
| --- | --- | --- | --- | --- | --- |
| 11. I feel privileged to care for ___ |  |  |  |  |  |
| 12. Others have dumped caring for ___ onto me |  |  |  |  |  |
| 13. My financial resources are adequate to pay for things that are required for caregiving |  |  |  |  |  |
| 14. My activities are centred around care for ___ |  |  |  |  |  |
| 15. Since caring for ___ it seems like I'm tired all of the time |  |  |  |  |  |
| 16. It is very difficult to get help from my family in taking care of ___ |  |  |  |  |  |
| 17. I resent having to take care of ___ |  |  |  |  |  |
| 18. I have to stop in the middle of work |  |  |  |  |  |
| 19. I really want to care for ___ |  |  |  |  |  |
| 20. My health has gotten worse since I've been caring for ___ |  |  |  |  |  |
| 21. I visit family and friends less since I have been caring for ___ |  |  |  |  |  |
| 22. I will never be able to do enough caregiving to repay ___ |  |  |  |  |  |
| 23. My family works together at caring for ___ |  |  |  |  |  |
| 24. I have eliminated things from my schedule since caring for ___ |  |  |  |  |  |
| 25. I have enough physical strength to care for ___ |  |  |  |  |  |
| 26. Since caring for ___ I feel my family has abandoned me |  |  |  |  |  |
| 27. Caring for ___ makes me feel good |  |  |  |  |  |
| 28. The constant interruptions make it difficult to find time for relaxation |  |  |  |  |  |
| 29. I am healthy enough to care for ___ |  |  |  |  |  |
| 30. Caring for ___ is important to me |  |  |  |  |  |
| 31. Caring for ___ has put a financial strain on the family |  |  |  |  |  |
| 32. My family (brothers, sisters, and children) left me alone to care for ___ |  |  |  |  |  |
| 33. I enjoy caring for ___ |  |  |  |  |  |
| 34. It's difficult to pay for ___'s health needs and services |  |  |  |  |  |

| I give care because: | Strongly Disagree | Somewhat Disagree | Somewhat Agree | Strongly Agree |
| --- | --- | --- | --- | --- |
| 35. It is my duty to provide care to elderly dependant family members |  |  |  |  |
| 36. It is important to set an example for the children in the family |  |  |  |  |
| 37. I was taught by my parents to care for elderly family members |  |  |  |  |
| 38. Of my religion and spiritual beliefs |  |  |  |  |
| 39. By giving care to elderly dependant family members, I give back what was given to me |  |  |  |  |
| 40. It strengthens the bond between me and them |  |  |  |  |
| 41. I was raised to believe care was provided in the family |  |  |  |  |
| 42. It is what my people have always done |  |  |  |  |
| 43. I feel as I am being useful and making a family contribution |  |  |  |  |
| 44. My family expect me to provide care |  |  |  |  |

*The next questions ask about changes you have experienced in your caregiving journey over time; please share any stories or examples of these experiences*

45. Has caring for X changed the type or amount of paid work that you are able to do?

46. Has being involved in caring for X changed your relationship with X?

47. Has caring for X changed your relationship with friends or members of your family?

Third Topic: Care and assistance

*The next questions ask about the types of care you provide, how often, and if anyone else helps you*

| Types of care and assistance | What care and assistance do YOU give to X? | How often do YOU give this assistance? | Is there additional assistance? If so,  Who? With what cares? How often? |
| --- | --- | --- | --- |
| 48. Personal care: eating, grooming, bathing, dressing, toileting, mobility |  |  |  |
| 49. Day-to-day assistance: phone calls, leisure, medication management, money management, transportation |  |  |  |
| 50. Household chores: shopping, preparing meals, laundry, light housework, maintenance |  |  |  |
| 51. Additional support: companionship, support with decisions, emotional and spiritual support, interpreting |  |  |  |

Fourth Topic: Health Services

*The next questions ask about goals, health providers and services you have experienced over time*

52. What are your goals in caring for X?

53. Who helps you to work towards those goals?

54. Can you tell me about your relationship with X’s general practitioner or practice nurse?

55. Do the GP and/or nurse understand what is important to you and X?

56. Do the GP / nurse support you in your role as a carer for X?

57. Are there any other health professionals who support you and X?

58. Can you tell me about your relationship with other health professionals?

59. Do other health professionals understand what is important to you and X?

60. Are there services that you currently don’t receive that could help you to care for X?

61. Can you give any examples of services that could improve in the way provide care?

62. Is there any advice that you could give to the people who provide these services?

Fifth Topic: Health and well-being

*The next questions ask about X’s general well-being, as in spiritual, mind, body and relationships*

|  | Much more | More | No change | Less | Much less |
| --- | --- | --- | --- | --- | --- |
| 63. As a result of primary health care does X feel:   1. more valued as a person 2. stronger in himself/herself as (ethnicity) 3. more content within himself/herself 4. healthier from a spiritual point of view |  |  |  |  |  |
| 64. As a result of primary health care does X feel:   1. more able to set goals for himself/herself 2. more able to think, feel & act in a positive manner 3. more able to manage unwelcome thoughts & feelings 4. more able to understand how to deal with his/her health problem |  |  |  |  |  |
| 65. As a result of primary health care does X feel:   1. more able to move about without pain or distress 2. more committed to having good physical health 3. more able to understand how physical health improves mental well-being 4. physically healthier |  |  |  |  |  |
| 66. As a result of primary health care does X feel:   1. more able to communicate with Whanau/family 2. more confident in his/her relationships with other people 3. clearer about the relationship with Whanau/family 4. more able to participate in the community |  |  |  |  |  |

Is there anything else that you would like to tell me?

*Thank you for your time and willingness to participate in this study.*
